# Unique gut microbiome signatures associated with physical and cognitive function

**DOI:** 10.1002/alz.092044

**Published:** 2025-01-09

**Authors:** Sridevi U Nair, Yi Lin, Shalini Jain, Diptaraj S Chaudhari, Rohit Shukla, Vivek Kumar, Robert T Mankowski, Hongdao Meng, Thomas W Buford, George A Kuchel, Hariom Yadav, MiaGB Consortium

**Affiliations:** ^1^ University of South Florida, Tampa, FL USA; ^2^ University of Florida, Gainesville, FL USA; ^3^ University of Alabama, Birmingham, AL USA; ^4^ University of Connecticut Medical Center, West Hartford, CT USA

## Abstract

**Background:**

Emerging studies suggest the interaction of the gut microbiome with physical and cognitive function. However, it remains unknown whether the same microbiome is linked with both functions and/or if they are unique.

**Method:**

Utilizing data and samples from the Microbiome in Aging Gut and Brain (MiaGB) Consortium cohort, we assessed the whole genome gut microbiome metagenomics in 113 older adults (mean age 72 years [39 male and 74 female]) with high and low physical functions (measured using Short Physical Performance Battery [SPPB]) and cognitive function (measure using Montreal Cognitive Assessment [MoCA]). Based on the SPPB score, participants were categorized into low (score: 3‐9) and high (score: 10‐12) physical function groups, while based on MoCA score, mild cognitive impaired (MCI; MoCA:18‐25) and cognitively healthy (MoCA: 26‐30).

**Result:**

We observed the presence of distinct and specific microbiome signatures that are uniquely associated with the low and high physical functioning groups, as opposed to those specific to cognitive functioning. Specifically, in low physical functioning group Bacteroidacea and Rikenellaceae were increased and Clostridiaceae were decreased compared to high physical functioning group. On the other hand, Streptococcaceae decreased in low cognitive function group compared to cognitively healthy people. Increase in the ratio of Firmicutes ‐Bacteroidetes was specific for physical activity, whereas the increase in the ratio of Actinobacteria‐Proteobacteria was specific for cognitive function.

**Conclusion:**

Differentiating unique and specific microbiome signatures for physical function and cognitive function represents significant progress in the field of the gut‐brain axis. Our results support the distinct impact of the microbiome to impact the gut‐muscle axis versus the gut‐brain axis; and opens the doors for further comprehensive studies to determine the causal role of the microbiome in physical and cognitive functions, as well as its potential to be a biomarker for predicting the risk of decline in these functions in older adults – two of the most debilitating public health problems in older adults.

